# Effects of once-weekly subcutaneous retatrutide on weight and metabolic markers: A systematic review and meta-analysis of randomized controlled trials

**DOI:** 10.1016/j.metop.2024.100321

**Published:** 2024-09-13

**Authors:** Eric Pasqualotto, Rafael Oliva Morgado Ferreira, Matheus Pedrotti Chavez, Alexandre Hohl, Marcelo Fernando Ronsoni, Tales Pasqualotto, Francisco Cezar Aquino de Moraes, Larissa Hespanhol, Janine Midori Figueiredo Watanabe, Carine Lütkemeyer, Simone van de Sande-Lee

**Affiliations:** a Department of Medicine, Federal University of Santa Catarina, Florianópolis, Brazil; b Department of Medicine, Universidade Alto Vale do Rio do Peixe, Caçador, Brazil; c Department of Medicine, Federal University of Pará, Belém, Brazil; d Department of Medicine, Federal University of Campina Grande, Campina Grande, Brazil; e Department of Medicine, State University of Piauí, Teresina, Brazil; f Independent Researcher, Florianópolis, SC, Brazil

**Keywords:** Retatrutide, Body weight, Obesity, Metabolism

## Abstract

**Aim:**

To assess the effects of once-weekly subcutaneous retatrutide on weight and metabolic markers and the occurrence of side effects in patients with overweight, obesity and/or type 2 diabetes (T2D).

**Methods:**

PubMed, Embase, Cochrane Library, and ClinicalTrials.gov databases were systematically searched for placebo-controlled, randomized clinical trials (RCTs) published up until February 23, 2024. Weighted mean differences (WMDs) for continuous outcomes and risk ratios (RRs) for binary endpoints were computed, with 95 % confidence intervals (CIs).

**Results:**

A total of three studies were included, comprising 640 patients, of whom 510 were prescribed retatrutide. Compared with placebo, retatrutide significantly reduced body weight (WMD -10.66 kg; 95 % CI -17.63, −3.69), body mass index (WMD -4.53 kg/m^2^; 95 % CI -7.51, −1.55), and waist circumference (WMD -6.61 cm; 95 % CI -13.17, −0.05). In addition, retatrutide significantly increased the proportion of patients who achieved a weight reduction of ≥5 % (RR 2.92; 95 % CI 2.17–3.93), ≥10 % (RR 9.32; 95 % CI 4.56–19.06), ≥15 % (RR 18.40; 95 % CI 6.00–56.42), and ≥20 % (RR 16.61; 95 % CI 4.17–66.12).

**Conclusions:**

In this meta-analysis, the use of once-weekly subcutaneous retatrutide was associated with a significant reduction in body weight and improvement of metabolic markers in patients with overweight, obesity and/or T2D, compared with placebo, with an increase in non-severe gastrointestinal and hypersensitivity adverse events. Phase 3 RCTs are expected to shed further light on the efficacy and safety of once-weekly subcutaneous retatrutide over the long term.

## Highlights

• The novel triple-hormone agonist retatrutide led to substantial weight reduction in patients with overweight, obesity and/or type 2 diabetes.
• Retatrutide was associated with a significant improvement of metabolic markers in patients with overweight, obesity, and/or T2D.
• Once-weekly subcutaneous retatrutide significantly increased non-severe gastrointestinal and hypersensitivity adverse events.

## Abbreviations

T2D: type 2 diabetes
GLP-1: glucagon-like peptide 1
RA: receptor agonist
GIP: gastric inhibitory polypeptide
GCG: glucagon
RCT: randomized controlled trial
PRISMA: Preferred Reporting Items for Systematic Reviews and Meta-Analysis
PROSPERO: International Prospective Register of Systematic Reviews
BMI: body mass index
HbA1c: glycated hemoglobin
SBP: systolic blood pressure
DBP: diastolic blood pressure
HDL: high-density lipoprotein
LDL: low-density lipoprotein
VLDL: very-low-density lipoprotein
AST: aspartate aminotransferase
ALT: alanine aminotransferase
TEAEs: treatment-emergent adverse events
Rob-2: Cochrane Collaboration tool for assessing risk of bias in randomized trials
GRADE: Grading of Recommendation, Assessment, Development and Evaluation
WMD: weighted mean difference
SMD: standardized mean difference
RR: risk ratio
CI: confidence interval

## 1 Introduction

Obesity presents as a multifaceted chronic condition affecting millions globally. According to the World Obesity Federation, in 2020, there were over 2.6 billion adults with overweight or obesity, and it is estimated that this number will increase to over 3 billion by 2025 and more than 4 billion in 2035 [1]. Numerous complications, including type 2 diabetes (T2D), hypertension, dyslipidemia, and cardiovascular disease, have been correlated with obesity, in addition to an elevated risk of overall mortality [2,3]. Of note, weight loss of 5 %–15 % of baseline body weight significantly reduces the risk of several complications in patients with overweight or obesity [4].

The recent generation of glucose-lowering agents, exemplified by glucagon-like peptide 1 (GLP-1) receptor agonists (RAs), has found application in numerous patients to achieve a significant weight reduction, glycemic control in T2D, and mitigation of cardiovascular risk [[5], [6], [7]]. Furthermore, concomitant agonism of other nutrient-stimulated hormones, such as gastric inhibitory polypeptide (GIP) and glucagon (GCG), increase the central anorectic effect [8]. Specifically, GIP agonism promotes lipolysis, while GCG agonism enhances substrate utilization and increases energy expenditure [9]. Consequently, investigational efforts have been directed towards single-, dual-, and triple-hormone receptor agonists as promising therapeutic modalities for weight reduction [5,[10], [11], [12]].

In this context, retatrutide, a new GIP/GLP-1/GCG RA administered once weekly, is currently under investigation for chronic weight management and associated complications [5,9,13]. Phase 1 and 2 trials have recently been published, addressing its efficacy and safety for reducing body weight and improving the glycemic profile of patients with T2D and/or obesity. A meta-analysis by Ayesh et al. demonstrated a significant effect of retatrutide on weight and glycated hemoglobina reduction compared to placebo or dulaglutide [14], however, our study represents the first comprehensive systematic review and meta-analysis of randomized controlled trials (RCTs) to provide pooled effect estimates regarding the efficacy and safety of once-weekly subcutaneous retatrutide, broadly evaluating its effect on weight and metabolic markers.

## 2 Methods

This systematic review followed the Preferred Reporting Items for Systematic Reviews and Meta-Analysis (PRISMA) guidelines [15]. The study protocol was registered in the International Prospective Register of Systematic Reviews (PROSPERO) with registration number CRD42023456900.

### 2.1 Search strategy and data extraction

PubMed, Embase, Cochrane Library, and ClinicalTrials.gov were systematically searched from inception to February 23, 2024, with the following search terms: Retatrutide OR LY3437943. Aiming the inclusion of additional studies, references of the included articles and systematic reviews of the literature were evaluated. Three authors (E.P., L.H., and M.P.C.) independently extracted baseline characteristics and data outcomes following predefined search criteria. Four authors resolved disagreements by consensus (E.P., L.H., M.P.C., and S.S.L).

### 2.2 Eligibility criteria

Studies with the following criteria were included: (1) RCTs; (2) comparing once-weekly subcutaneous retatrutide and placebo; (3) comprising adult patients (≥18 years) with overweight, obesity and/or T2D; and (4) reporting at least one of the outcomes of interest. Studies with the following criteria were excluded: (1) RCTs with recruiting status or without results; and (2) overlapping population.

### 2.3 Endpoints and subgroup analysis

Outcomes of interest were: body weight, body mass index (BMI), weight reduction of ≥5 %, weight reduction of ≥10 %, weight reduction of ≥15 %, weight reduction of ≥20 %, waist circumference, daily mean blood glucose, glycated hemoglobin (HbA1c), fasting glucose, fasting insulin, fasting C-peptide, glucagon, systolic blood pressure (SBP), diastolic blood pressure (DBP), high-density lipoprotein (HDL), low-density lipoprotein (LDL), very-low-density lipoprotein (VLDL), triglycerides, aspartate aminotransferase (AST), alanine aminotransferase (ALT), amylase, lipase, treatment-emergent adverse events (TEAEs), serious adverse events, pulse rate, deaths, TEAEs leading to study treatment discontinuation, diarrhea, nausea, vomiting, constipation, abdominal pain, dyspepsia, gastroesophageal reflux, headache, dizziness, hypersensitivity, hepatic or biliary disease, severe gastrointestinal adverse events, major adverse cardiovascular events, injection site reaction, cardiac arrhythmias. Daily mean blood glucose was obtained from a 6-point self-monitored blood glucose profile.

A post hoc subgroup analyses were performed with patients with T2D.

### 2.4 Risk of bias assessment

The Cochrane Collaboration tool for assessing risk of bias in randomized trials (Rob-2) was used to assess the quality of individual RCTs [16]. Each trial received a high, low, or unclear risk of bias score in five domains: randomization process; deviations from the intended interventions; missing outcomes; measurement of the outcome; and selection of reported results. Two independent authors conducted the risk of bias assessment (E.P. and R.O.M.F.) and disagreements were resolved unanimously with the senior author (S.S.L.).

### 2.5 Quality assessment

The quality of evidence was assessed according to the Grading of Recommendation, Assessment, Development and Evaluation (GRADE) guidelines [17]. Very low, low, moderate, or high-quality evidence grades were designed for the outcomes based on the risk of bias, inconsistency of results, imprecision, publication bias, and magnitude of treatment effects [18].

### 2.6 Data blending and conversions

Data conversions and the combination of means and standard deviations were conducted using the Review Manager 5.4 (Nordic Cochrane Centre, The Cochrane Collaboration, Copenhagen, Denmark) calculator, in accordance with the guidelines outlined in the Cochrane Handbook for Systematic Reviews of Interventions [19]. Specifically, where necessary, we converted data into a consistent format to ensure appropriate statistical comparisons.

For the analysis, data from the 0.5 mg, 1 mg, 1.5 mg, 3 mg, 4 mg, 8 mg, and 12 mg doses of retatrutide were included. Additionally, data from groups receiving escalated doses of retatrutide were also aggregated and considered in the statistical analysis to provide a comprehensive assessment of the treatment effect across all dosing regimens.

### 2.7 Statistical analysis

The treatment effects for continuous outcomes were compared using weighted mean differences (WMDs) or standardized mean differences (SMDs) and binary endpoints were evaluated using risk ratios (RRs), with 95 % confidence intervals (CIs). Heterogeneity was assessed with the Cochran Q-test and I^2^ statistics; P values < 0.10 and I^2^ values > 25 % were considered to indicate significance for heterogeneity [20]. DerSimonian and Laird random-effects models were used for all endpoints [21]. Statistical analyses were performed using R statistical software, version 4.2.3 (R Foundation for Statistical Computing).

### 2.8 Sensitivity analysis

Leave-one-out procedures were used to identify influential studies and their effect on the pooled estimates, evaluating the heterogeneity. This procedure was carried out by removing data from one study and reanalyzing the remaining data. When pooled effect size p-values changed from significant to non-significant, or vice-versa, study dominance was assigned.

## 3 Results

### 3.1 Study selection and characteristics

**Fig. 1:**
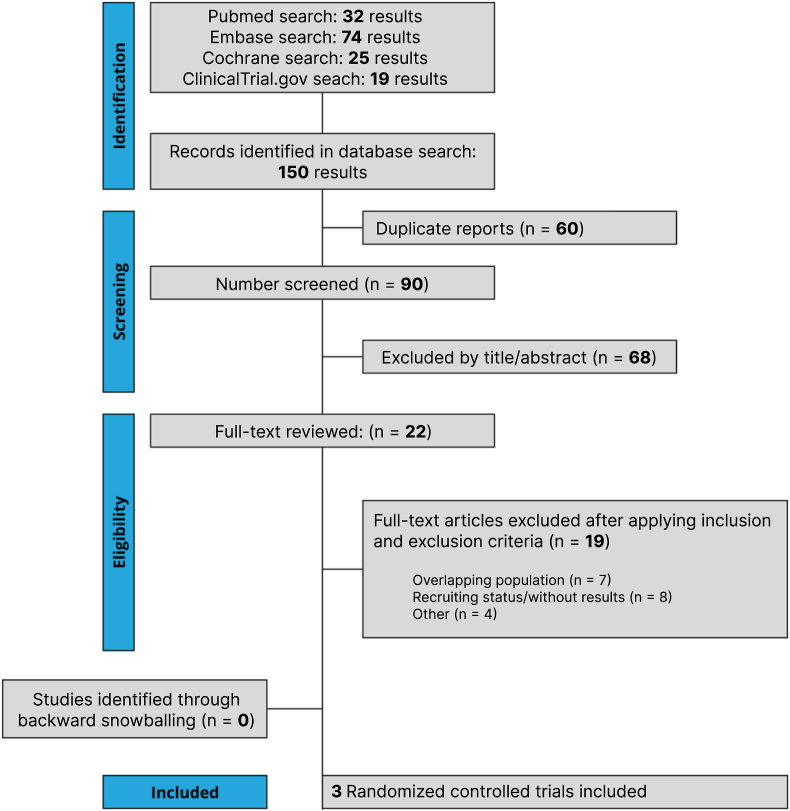
Preferred Reporting Items for Systematic Review and Meta-Analysis (PRISMA) flow diagram of study screening and selection.

The initial search yielded 150 results, as detailed in Fig. 1. After removal of duplicate records and assessment of the studies based on title and abstract, 24 full-text studies remained for full review according to prespecified criteria. Of these, three RCTs were included, comprising 640 patients [5,9,13]. A total of 510 patients were randomized to the retatrutide group, while 130 were to placebo. The mean age was 52.23 years. The follow-up ranged from 16 to 48 weeks. Study and participant characteristics are detailed in Table 1.

**Table 1:** Design and characteristics of studies included in the meta-analysis.

Study	Rosenstock 2023	Urva 2022	Jastreboff 2023
Trial phase	Phase 2	Phase 1b	Phase 2
Follow-up	36 weeks	16 weeks	48 weeks
Inclusion criteria	T2D and BMI of 25–50 kg/m^2^	T2D, BMI of 23–50 kg/m^2^, and stable bodyweight^a^	BMI of ≥30 kg/m^2^ or BMI of 27–30 kg/m^2^ plus at least one weight-related condition
Retatrutide doses	0.5 mg, 4 mg group, 4 mg escalation group [from 2 mg to 4 mg], 8 mg slow escalation group [from 2 mg to 4 mg–8 mg], 8 mg fast escalation group [from 4 mg to 8 mg], or 12 mg escalation group [from 2 mg to 4 mg–8 mg to 12 mg]	0.5 mg, 1.5 mg, 3 mg, 3/6 mg [from 3 mg to 6 mg], and 3/6/9/12 mg [from 3 mg to 6 mg–9 mg to 12 mg]	1 mg, 4 mg [initial dose 2 mg], 4 mg [initial dose 4 mg], 8 mg [initial dose 2 mg], 8 mg [initial dose 4 mg], or 12 mg [initial dose 2 mg]
Sample sizes, n (%) RG/PG	190 (67.6)/45 (16)	52 (72.2)/15 (20)	268 (79.3)/70 (20.7)
Age, years RG/PG	56.1 (9.18)/57.6 (10.8)	58.088 (7.760)/58.8 (6.4)	48.19 (12.8)/48.0 (12.5)
Male, n (%) RG/PG	90 (47.3)/22 (49)	28 (38.8)/3 (20)	139 (51.86)/36 (51.4)
BMI, kg/m^2^ RG/PG	34.9 (6.3)/33.8 (4.9)	32.2 (4.9)/32.3 (6.2)	37.36 (5.7)/37.3 (5.9)
Body weight, kg RG/PG	98.5 (21.5)/94.6 (16.6)	86.2 (16.8)/84.1 (19.9)	107.35 (21.4)/109.2 (20.9)
Waist circumference, cm RG/PG	111.7 (16.26)/108.6 (12.3)	106.2 (10.82)/105.8 (17.6)	115.6 (14.9)/115.1 (13.9)
HbA1c, % RG/PG	8.2 (1.08)/8.4 (1.1)	8.6 (0.89)/8.83 (1.06)	NA/NA
Fasting serum glucose, mmol/L RG/PG	9.4 (2.9)/10.2 (3.4)	NA/NA	NA/NA
Systolic blood pressure, mmHg RG/PG	129.2 (12.5)/131.9 (15)	NA/NA	NA/NA
Diastolic blood pressure, mmHg RG/PG	79.6 (8.5)/78.6 (9.8)	NA/NA	NA/NA
Total cholesterol, mg/dL RG/PG	178.5 (25.6)/164.1 (31.6)	NA/NA	NA/NA
HDL cholesterol, mg/dL RG/PG	42.9 (26.6)/44.3 (28.7)	NA/NA	NA/NA
Non-HDL cholesterol, mg/dL RG/PG	132.7 (31.7)/117.2 (40.3)	NA/NA	NA/NA
Triglycerides, mg/dL RG/PG	166.5 (62.5)/143.7 (54.8)	NA/NA	NA/NA
Duration of obesity, years RG/PG	NA/NA	NA/NA	13.36 (11.12)/11.7 (9.3)
Duration of diabetes, years RG/PG	NA/NA	10.6 (5.7)/9.2 (6.0)	NA/NA
Metformin use, n (%)	132.2 (69.8)/35 (78)	NA/NA	NA/NA
Prediabetes, n (%)	NA/NA	NA/NA	97 (36)/26 (37)
Hypertension, n (%)	NA/NA	NA/NA	104 (38)/40 (57)
Dyslipidemia, n (%)	NA/NA	NA/NA	87 (32.4)/23 (33)

Data are presented as mean (SD) or n (%).

a Stable body weight was defined as <5 % change over the past 3 months. BMI, body mass index; HbA1c, glycated hemoglobin; HDL, high-density lipoprotein; NA, not available; PG, placebo group; RG, retatrutide group; SD, standard deviation; T2D, type 2 diabetes.

### 3.2 Pooled analysis of all studies

#### 3.2.1 Weight reduction and metabolic markers

**Fig. 2:**
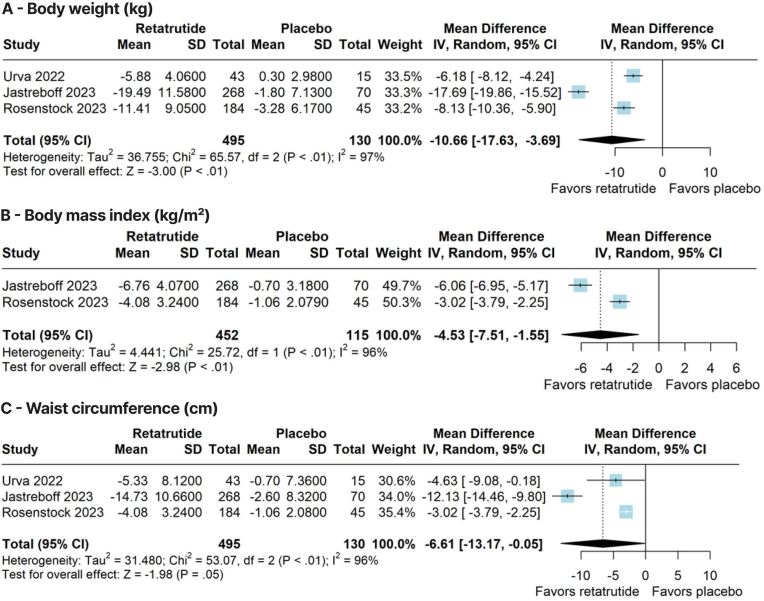
Forest plots of pooled comparisons between retatrutide and placebo. **(A)** Body weight (kg). **(B)** Body mass index (kg/m^2^). **(C)** Waist circumference (cm).

**Fig. 3:**
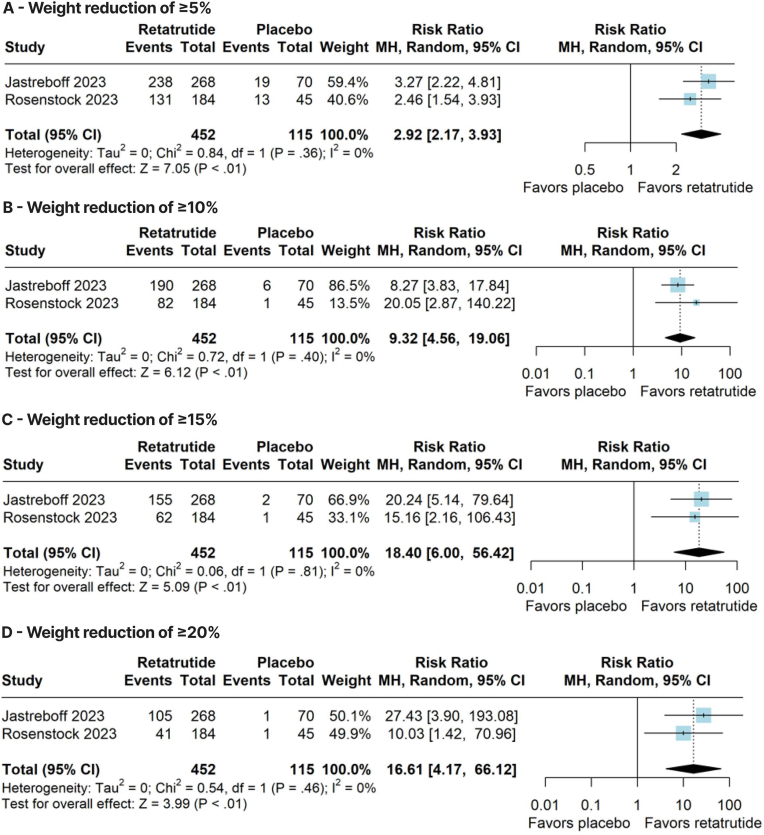
Forest plots of pooled comparisons between retatrutide and placebo. **(A)** Weight reduction of ≥5 %. **(B)** Weight reduction of ≥10 %. **(C)** Weight reduction of ≥15 %. **(D)** Weight reduction of ≥20 %.

Compared with placebo, retatrutide significantly reduced body weight (WMD –10.66 kg; 95 % CI –17.63, −3.69; p < 0.01; I^2^ = 97 %; Fig. 2A), BMI (WMD –4.53 kg/m^2^; 95 % CI –7.51, −1.55; p < 0.01; I^2^ = 96 %; Fig. 2B), and waist circumference (WMD –6.61 cm; 95 % CI –13.17, −0.05; p = 0.05; I^2^ = 96 %; Fig. 2C). In addition, retatrutide significantly increased the proportion of patients who achieved a weight reduction of ≥5 % (RR 2.92; 95 % CI 2.17–3.93; p < 0.01; I^2^ = 0 %; Fig. 3A), ≥10 % (RR 9.32; 95 % CI 4.56–19.06; p < 0.01; I^2^ = 0 %; Fig. 3B), ≥15 % (RR 18.40; 95 % CI 6.00–56.42; p < 0.01; I^2^ = 0 %; Fig. 3C), and ≥20 % (RR 16.61; 95 % CI 4.17–66.12; p < 0.01; I^2^ = 0 %; Fig. 3D).

**Fig. 4:**
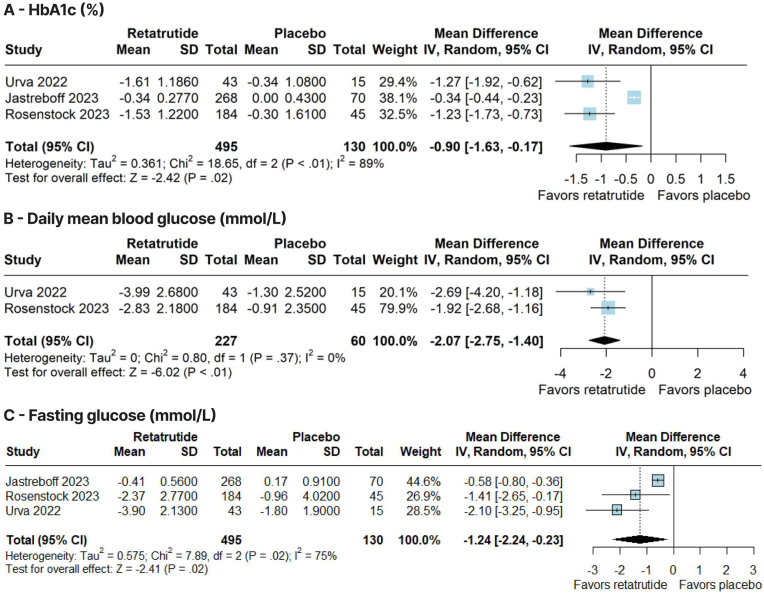
Forest plots of pooled comparisons between retatrutide and placebo. **(A)** Glycated hemoglobin (HbA1c) (%). **(B)** Daily mean blood glucose (mmol/L). **(C)** Fasting glucose (mmol/L).

Also, there was a significant reduction in HbA1c (WMD –0.90 %; 95 % CI –1.63, −0.17; p = 0.02; I^2^ = 89 %; Fig. 4A), daily mean blood glucose (WMD –2.07 mmol/L; 95 % CI –2.75, −1.40; p < 0.01; I^2^ = 0 %; Fig. 4B), fasting glucose (WMD –1.24 mmol/L; 95 % CI –2.24, −0.23; p = 0.02; I^2^ = 75 %; Fig. 4C), and glucagon levels (SMD –1.40; 95 % CI –2.80, −0.01; p = 0.05; I^2^ = 92 %; Supplementary Material 1, Fig. S1A) in favor of the retatrutide group, compared with placebo. There was a significant increase in fasting insulin in the retatrutide group (SMD 0.29; 95 % CI 0.08, 0.49; p < 0.01; I^2^ = 0 %; Supplementary Material 1, Fig. S1B). However, there was no significant difference between groups in fasting C-peptide (SMD –0.01; 95 % CI –0.29, 0.28; p = 0.95; I^2^ = 0 %; Supplementary Material 1, Fig. S1C). In the subgroup analysis for patients with T2D, retatrutide significantly reduced HbA1c (WMD –1.24 %; 95 % CI –1.64, −0.85; p < 0.01; I^2^ = 0 %), daily mean blood glucose (Fig. 4B), and fasting glucose (WMD –1.78 mmol/L; 95 % CI –2.62, −0.94; p < 0.01; I^2^ = 0 %) compared with placebo.

There was a significant reduction in SBP (WMD –4.70 mmHg; 95 % CI –8.56, −0.83; p = 0.02; I^2^ = 54 %; Supplementary Material 1, Fig. S2A) and DBP (WMD –1.73 mmHg; 95 % CI –3.04, −0.42; p < 0.01; I^2^ = 0 %; Supplementary Material 1, Fig. S2B). However, there was no significant difference between groups in pulse rate (WMD 2.65 bpm; 95 % CI –0.95, 6.25; p = 0.15; I^2^ = 79 %; Supplementary Material 1, Fig. S2C).

In addition, there was a significant reduction in VLDL (WMD –22.74 %; 95 % CI –36.81, −8.67; p < 0.01; I^2^ = 71 %; Supplementary Material 1, Fig. S3A), AST (WMD –2.72 U/L; 95 % CI –4.14, −1.29; p < 0.01; I^2^ = 0 %; Supplementary Material 1, Fig. S4A). There was a significant increase in amylase in the retatrutide group (SMD 0.29; 95 % CI 0.08, 0.49; p < 0.01; I^2^ = 0 %; Supplementary Material 1, Fig. S4B). Nevertheless, there was no significant difference between groups in HDL (WMD 3.57 %; 95 % CI –7.10, 14.24; p = 0.51; I^2^ = 89 %; Supplementary Material 1, Fig. S3B), LDL (WMD 2.65 %; 95 % CI –0.95, 6.25; p = 0.15; I^2^ = 79 %; Supplementary Material 1, Fig. S3C), triglycerides (WMD –8.63 %; 95 % CI –38.10, 20.85; p = 0.57; I^2^ = 92 %; Supplementary Material 1, Fig. S3D), ALT (WMD –2.15 U/L; 95 % CI –5.63, 1.34; p = 0.23; I^2^ = 68 %; Supplementary Material 1, Fig. S4C), and lipase (SMD 0.27; 95 % CI –0.22, 0.77; p = 0.28; I^2^ = 82 %; Supplementary Material 1, Fig. S4D).

#### 3.2.2 Safety

Compared with placebo, retatrutide significantly increased the rate of TEAEs (RR 1.18; 95 % CI 1.04–1.35; p = 0.01; I^2^ = 0 %; Supplementary Material 1, Fig. S5A), nausea (RR 2.68; 95 % CI 1.54–4.68; p < 0.01; I^2^ = 0 %; Supplementary Material 1, Fig. S5B), vomiting (RR 4.59; 95 % CI 1.30–16.24; p = 0.02; I^2^ = 0 %; Supplementary Material 1, Fig. S5C), constipation (RR 3.08; 95 % CI 1.12–8.45; p = 0.03; I^2^ = 0 %; Supplementary Material 1, Fig. S5D), and hypersensitivity (RR 3.79; 95 % CI 1.20–11.96; p = 0.02; I^2^ = 0 %; Supplementary Material 1, Fig. S6A).

There was no significant difference between groups for serious adverse events (RR 1.46; 95 % CI 0.46–4.61; p = 0.52 I^2^ = 49 %; Supplementary Material 1, Fig. S6B), TEAEs leading to study treatment discontinuation (RR 2.87; 95 % CI 0.90–9.21; p = 0.08; I^2^ = 0 %; Supplementary Material 1, Fig. S6C), diarrhea (RR 1.56; 95 % CI 0.87–2.78; p = 0.13; I^2^ = 0 %; Supplementary Material 1, Fig. S6D), abdominal pain (RR 1.16; 95 % CI 0.33–4.04; p = 0.82; I^2^ = 0 %; Supplementary Material 1, Fig. S7A), pancreatitis (RR 0.99; 95 % CI 0.11–8.87; p = 0.99; I^2^ = 0 %; Supplementary Material 1, Fig. S7B), eructation (RR 3.23; 95 % CI 0.43–24.19; p = 0.25; I^2^ = 0 %; Supplementary Material 1, Fig. S7C), dyspepsia (RR 1.60; 95 % CI 0.53–4.88; p = 0.41; I^2^ = 0 %; Supplementary Material 1, Fig. S7D), gastroesophageal reflux (RR 2.32; 95 % CI 0.42–12.68; p = 0.33; I^2^ = 0 %; Supplementary Material 1, Fig. S8A), hepatic or biliary disease (RR 1.38; 95 % CI 0.41–4.70; p = 0.60; I^2^ = 0 %; Supplementary Material 1, Fig. S8B), severe gastrointestinal adverse events (RR 2.26; 95 % CI 0.28–17.99; p = 0.44; I^2^ = 0 %; Supplementary Material 1, Fig. S8C), headache (RR 1.75; 95 % CI 0.34–9.14; p = 0.50; I^2^ = 24 %; Supplementary Material 1, Fig. S8D), dizziness (RR 1.81; 95 % CI 0.55–5.97; p = 0.33; I^2^ = 0 %; Supplementary Material 1, Fig. S9A), cardiac arrhythmias (RR 3.41; 95 % CI 0.86–13.55; p = 0.08; I^2^ = 29 %; Supplementary Material 1, Fig. S9B), major adverse cardiovascular events (RR 0.99; 95 % CI 0.11–8.91; p = 1.00; I^2^ = 0 %; Supplementary Material 1, Fig. S9C), injection site reaction (RR 1.12; 95 % CI 0.11–11.48; p = 0.92; I^2^ = 52 %; Supplementary Material 1, Fig. S9D), and deaths (RR 0.28; 95 % CI 0.03–2.63; p = 0.26; I^2^ = 0 %; Supplementary Material 1, Fig. S9E).

### 3.3 Sensitivity analysis

We performed a leave-one-out sensitivity analysis for the body weight outcome. The outcome showed stability, without major changes in significance with the removal of each individual study. The leave-one-out sensitivity analysis plot is detailed in Supplementary Material 1, Fig. S10.

### 3.4 Risk of bias and quality assessment

The individual appraisal of each RCT included in this meta-analysis is outlined in Supplementary Material 1, Fig. S11. Overall, all studies were deemed at low risk of bias [5,9,13].

According to the GRADE assessment, low-quality evidence was assigned for the outcomes of body weight, BMI, and HbA1c. Moderate-quality evidence was assigned for the weight reduction of ≥5 % outcome. Meanwhile, high-quality evidence was assigned for the outcomes of weight reduction of ≥10 %, weight reduction of ≥15 %, and weight reduction of ≥20 %. The main domains responsible for reducing the quality of evidence of the outcomes were: inconsistency of results due to heterogeneity, and imprecision due to the small number of RCTs included in the statistical analysis. Quality assessment is detailed in Supplementary Material 2.

## 4 Discussion

In this systematic review and meta-analysis of 3 RCTs involving 640 patients with overweight, obesity and/or T2D, we assessed the efficacy and safety of once-weekly retatrutide compared with placebo. Our key findings were as follows: (1) retatrutide significantly reduced body weight, BMI, and waist circumference; (2) retatrutide was associated with a significantly higher proportion of patients achieving weight loss of >5 %, >10 %, >15 %, and >20 %; (3) retatrutide significantly reduced HbA1c and daily mean blood glucose; (4) retatrutide significantly increased gastrointestinal-related adverse events and hypersensitivity events; and (5) retatrutide did not increase serious adverse events.

GLP-1 RAs are progressively becoming integrated into the treatment of obesity or overweight in conjunction with lifestyle modifications [22]. Recently introduced as a pharmacotherapeutic intervention for obesity, semaglutide 2.4 mg has manifested a notable placebo-adjusted weight reduction of 12.4 %, with almost one-third of participants achieving a substantial weight loss of 20 % or more [23,24]. Furthermore, tirzepatide, characterized as a dual agonist targeting GIP and GLP-1, has exhibited efficacy in weight reduction and recently secured approval from the Food and Drug Administration for managing both T2D and obesity [22,25,26]. The findings from the SURMOUNT-1 trial, which included individuals with obesity but without diabetes, demonstrated that weekly doses of tirzepatide at 5 mg, 10 mg, and 15 mg led to an average weight loss of 15 %, 19 %, and 21 %, respectively, in contrast to a mere 3 % observed in the placebo group over 72 weeks [24]. Pharmacotherapeutics based on nutrient-stimulated hormones aim to influence endogenous mechanisms governing body-fat mass and energy homeostasis [27]. Thus, it was hypothesized that the efficacy of GLP-1 agonism or GIP–GLP-1 agonism could be heightened when combined with GCG receptor activation, potentially amplifying impacts on energy intake, substrate utilization, and energy expenditure [5,12].

In a phase 1b trial that included participants with T2D, administration of retatrutide led to a placebo-adjusted least-squares mean weight reduction of 8.96 kg in the 12 mg group after 12 weeks [9]. Furthermore, in a phase 2 trial involving patients with obesity, the least squares mean percentage change in body weight at 24 weeks was 17.5 % for retatrutide 12 mg, compared with 1.6 % in the placebo group, and at 48 weeks it was 24.2 % for retatrutide 12 mg, compared with 2.1 % in the placebo group. In this same phase 2 trial, weight loss of ≥5 %, ≥10 %, and ≥15 % were achieved respectively by 100 %, 93 %, and 83 % of patients treated with retatrutide 12 mg, at 48 weeks [5]. In our pooled analysis, a significant reduction in body weight was shown in favor of retatrutide, with an average reduction of 10.66 kg. In addition, retatrutide was associated with a significant increase in the proportion of patients achieving a clinically relevant weight reduction. Similar results were reported in previous meta-analyses with weekly subcutaneous semaglutide and once-daily oral semaglutide [23,28]. Of note, the phase 1 and 2 RCTs included in this meta-analysis had short follow-ups, and the weight curves indicate that a plateau in weight loss was not reached, suggesting that greater percentages of weight loss may be observed in studies with longer follow-ups. Furthermore, it is important to consider that our meta-analysis included individuals with T2D, a population in which the effect of anti-obesity medications is typically smaller than in patients without T2D.

Enthusiasm regarding retatrutide has arisen due to the potential efficacy of this drug relative to other interventions. The magnitude of initial weight loss reported in trials with retatrutide approached the ones seen after bariatric surgery, such as in Roux-en-Y gastric bypass surgery, in which an average net weight loss of 35 % was reported by a meta-analysis during the first few post-surgical years [29]. Furthermore, retatrutide significantly reduced glycemic markers. In a phase 1b study including patients with T2D, there was a 1.59 % reduction in HbA1c from baseline at 12 weeks in the group treated with retatrutide 12 mg [9]. In another phase 2 trial comprising patients with T2D, retatrutide 12 mg reduced HbA1c by 2.02 % from baseline at 24 weeks [13]. Furthermore, in patients with obesity and without T2D, a phase 2 trial demonstrated a least squares means a reduction of 0.4 % in HbA1c with retatrutide 12 mg [5]. In all trials, HbA1c reductions from baseline were observed in all retatrutide groups [5,9,13]. Accordingly, our meta-analysis showed a significant reduction in HbA1c, daily mean blood glucose, and fasting glucose in the retatrutide group, compared with placebo. Furthermore, subgroup analysis for patients with T2D demonstrated an even greater reduction in HbA1c and fasting glucose.

GLP-1 RAs have demonstrated cardiovascular benefits in patients with T2D, and the recent SELECT trial marked a significant milestone by showcasing, for the first time, a reduction in the composite outcome of cardiovascular mortality, nonfatal myocardial infarction, or nonfatal stroke with weekly subcutaneous semaglutide in individuals with overweight or obesity and cardiovascular disease, without diabetes [[30], [31], [32], [33]]. Additionally, tirzepatide exhibited a substantial reduction in major adverse cardiovascular events and cardiovascular death compared to placebo in a pooled analyses of the SURMOUNT-1 and SURPASS trials [34]. The effects of other GLP-1 RAs on cardiovascular outcomes remain to be evaluated in patients with obesity or overweight, however, the results of the SELECT trial highlight the importance of treating obesity to reduce cardiovascular risk [33]. In our meta-analysis, there was a significant reduction in SBP and DBP, in addition to a non-significant difference between groups in major adverse cardiovascular events.

In a previous network meta-analysis assessing approved drugs for the treatment of overweight and obesity, it was observed that GLP-1 analogs (semaglutide and liraglutide) might induce adverse effects resulting in treatment discontinuation [35]. Notably, drugs associated with the greatest risk of adverse events leading to discontinuation of treatment included phentermine-topiramate and naltrexone-bupropion [35]. Additionally, a comparison between daily and weekly regimens of semaglutide and liraglutide revealed that the former had higher withdrawal rates due to adverse events when contrasted with a placebo [6]. In our meta-analysis, a higher rate of gastrointestinal-related adverse events, particularly nausea, vomiting, and constipation, in addition to hypersensitivity events was found in patients treated with retatrutide. However, it was reassuring to note that there was no significant increase in serious adverse events.

This study has limitations. First, the analysis was based on a limited number of phase 1 and 2 RCTs, different retatrutide doses and populations, which may influence the effect size found in our results. Second, there was moderate to high heterogeneity in some of the outcomes analyzed. Third, RCTs evaluated in this meta-analysis presented different inclusion criteria, which may influence our results. The high heterogeneity observed in the meta-analysis is probably related to differences in the populations evaluated in the studies, which was represented in the subgroup analysis for patients with T2D, in which heterogeneity was 0 % with the homogeneous population. Due to the limited number of studies included, it was not possible to perform more robust meta-regressions or subgroup analyses. Thus, we highlight population variability as a possible source of heterogeneity, underscoring the importance of considering this factor in the interpretation of the overall findings. We performed a leave-one-out sensitivity analysis as an alternative to this and found consistent results after the removal of each study from the analysis. Fourth, while our meta-analysis aggregated all doses of retatrutide into a single global analysis, we did not evaluate the effects of specific dosages. As such, we cannot make definitive conclusions about potential differences in efficacy or side effects between different doses. This represents a key limitation of our study, as there may be variations in clinical outcomes and tolerance across the dose spectrum that our analysis could not detect. Clinicians should remain cautious when interpreting these findings and consider that lower or higher doses of retatrutide may present distinct trade-offs in terms of both efficacy and side effects. Finally, although this study represents the largest pooled analysis of patients treated with retatrutide, it remains underpowered to endpoints of metabolic, cardiovascular, and clinical effects.

In conclusion, in this meta-analysis, the use of once-weekly subcutaneous retatrutide was associated with a significant reduction in body weight and improvement of metabolic markers in patients with overweight, obesity and/or T2D, compared with placebo, with an increase in non-severe gastrointestinal and hypersensitivity adverse events. The low to high quality evidence of the results and limitations should be considered. Phase 3 RCTs are expected to shed further light on the efficacy and safety of once-weekly subcutaneous retatrutide over the long term.

## Conflict of interest

The authors declare no conflicts of interest.

## CRediT authorship contribution statement

**Eric Pasqualotto:** Writing – review & editing, Writing – original draft, Visualization, Validation, Supervision, Software, Resources, Project administration, Methodology, Investigation, Formal analysis, Data curation, Conceptualization. **Rafael Oliva Morgado Ferreira:** Writing – review & editing, Writing – original draft, Resources, Methodology, Formal analysis, Data curation. **Matheus Pedrotti Chavez:** Writing – review & editing, Writing – original draft, Resources, Methodology, Formal analysis, Data curation. **Alexandre Hohl:** Writing – review & editing, Writing – original draft, Resources, Methodology, Formal analysis, Data curation. **Marcelo Fernando Ronsoni:** Writing – review & editing, Writing – original draft, Resources, Methodology, Formal analysis, Data curation. **Tales Pasqualotto:** Writing – review & editing, Writing – original draft, Resources, Methodology, Formal analysis, Data curation. **Francisco Cezar Aquino de Moraes:** Writing – review & editing, Writing – original draft, Resources, Data curation. **Larissa Hespanhol:** Writing – review & editing, Writing – original draft, Resources, Data curation. **Janine Midori Figueiredo Watanabe:** Writing – review & editing, Writing – original draft. **Carine Lütkemeyer:** Writing – review & editing, Writing – original draft. **Simone van de Sande-Lee:** Writing – review & editing, Writing – original draft, Visualization, Validation, Supervision, Resources, Project administration, Methodology, Investigation, Formal analysis, Data curation, Conceptualization.
