# Supplementary material for: Effects of once-weekly subcutaneous retatrutide on weight and metabolic markers: A systematic review and meta-analysis of randomized controlled trials
Source: Metabol Open. 2024 Sep 13;24:100321. doi: 10.1016/j.metop.2024.100321 (PMC11420505; doi:10.1016/j.metop.2024.100321)
Supplement: Multimedia component 1 [file mmc1.docx]

**Supplementary Material 1**

**Figure S1.** Forest plots of pooled comparisons between retatrutide and placebo. **(A)** Glucagon level. **(B)** Fasting insulin. **(C)** Fasting C-peptide.

**Figure S2.** Forest plots of pooled comparisons between retatrutide and placebo. **(A)** Systolic blood pressure (SBP) (mmHg). **(B)** Diastolic blood pressure (DBP) (mmHg). **(C)** Pulse rate (bpm).

**Figure S3.** Forest plots of pooled comparisons between retatrutide and placebo. **(A)** Very-low-density lipoprotein (VLDL) (%). **(B)** High-density lipoprotein (HDL) (%). **(C)** Low-density lipoprotein (%). **(D)** Triglycerides (%).

**Figure S4.** Forest plots of pooled comparisons between retatrutide and placebo. **(A)** Aspartate aminotransferase (AST) (U/L). **(B)** Amylase. **(C)** Alanine aminotransferase (ALT) (U/L). **(D)** Lipase.

**Figure S5.** Forest plots of pooled comparisons between retatrutide and placebo. **(A)** Treatment-emergent adverse events (TEAEs). **(B)** Nausea. **(C)** Vomiting. **(D)** Constipation.

**Figure S6.** Forest plots of pooled comparisons between retatrutide and placebo. **(A)** Hypersensitivity. **(B)** Serious adverse events. **(C)** Treatment-emergent adverse events (TEAEs) leading to study treatment discontinuation. **(D)** Diarrhea.

**Figure S7.** Forest plots of pooled comparisons between retatrutide and placebo. **(A)** Abdominal pain. **(B)** Pancreatitis. **(C)** Eructation. **(D)** Dyspepsia.

**Figure S8.** Forest plots of pooled comparisons between retatrutide and placebo. **(A)** Gastroesophageal reflux. **(B)** Hepatic or biliary disease. **(C)** Severe gastrointestinal adverse events. **(D)** Headache.

**Figure S9.** Forest plots of pooled comparisons between retatrutide and placebo. **(A)** Dizziness. **(B)** Cardiac arrhythmias. **(C)** Major adverse cardiovascular events. **(D)** Injection site reaction. **(E)** Deaths.

**Figure S10.** Leave-one-out sensitivity analysis plot for the body weight outcome.

**Figure S11.** Critical appraisal of randomized controlled trials according to the Cochrane Collaboration tool for assessing risk of bias in randomized trials.

**
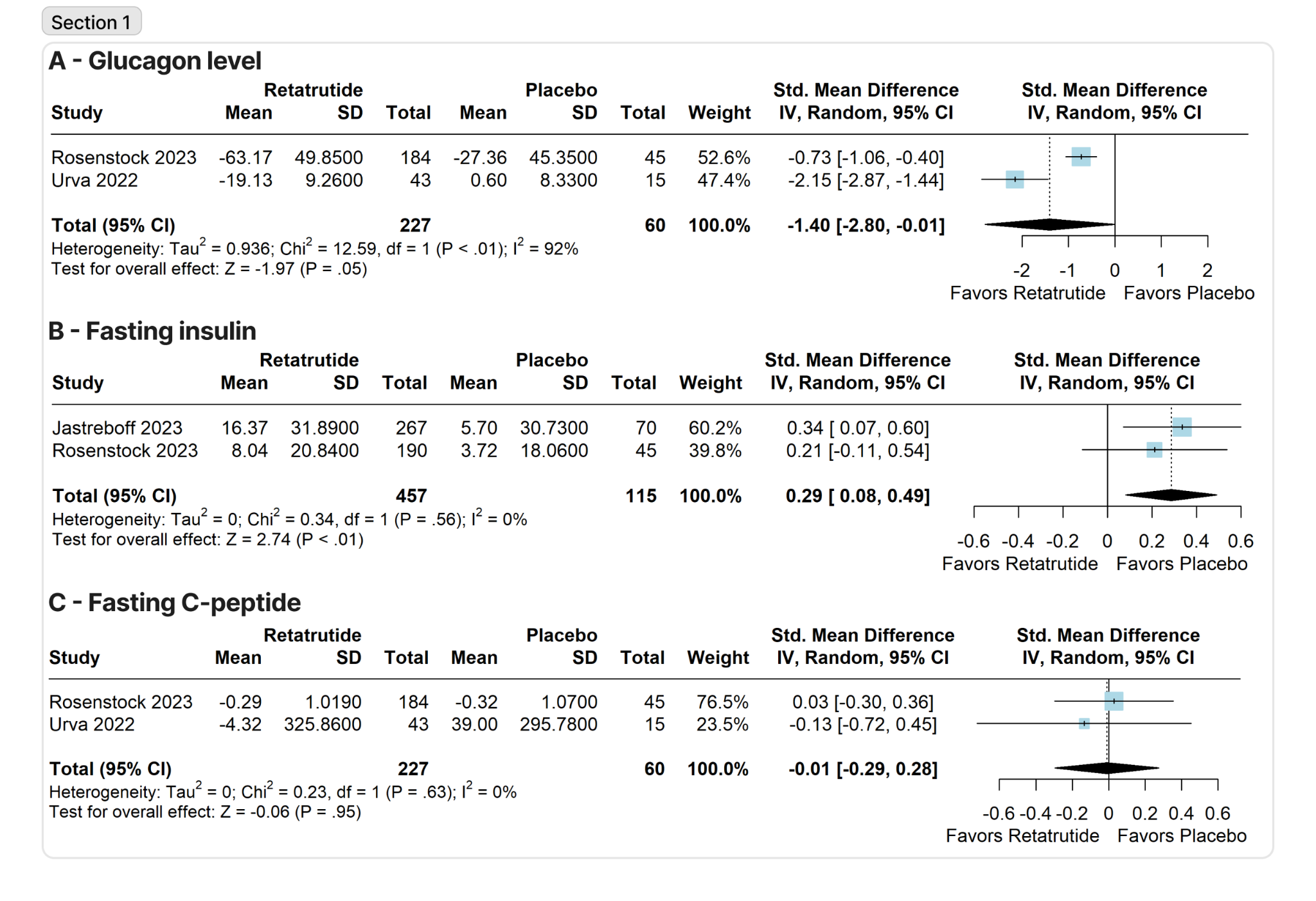
**

**Figure S1.** Forest plots of pooled comparisons between retatrutide and placebo. **(A)** Glucagon level. **(B)** Fasting insulin. **(C)** Fasting C-peptide.

**
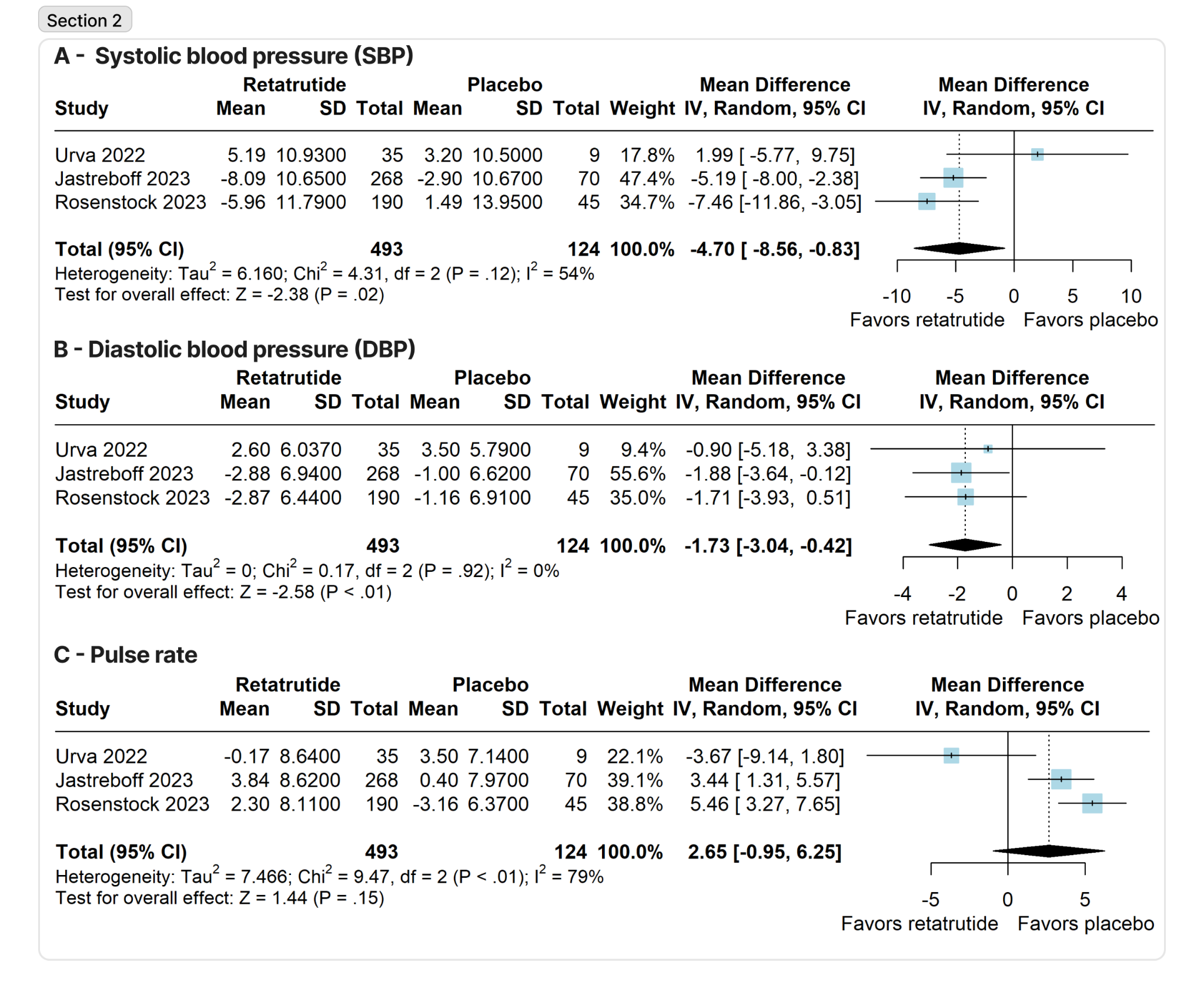
**

**Figure S2.** Forest plots of pooled comparisons between retatrutide and placebo. **(A)** Systolic blood pressure (SBP) (mmHg). **(B)** Diastolic blood pressure (DBP) (mmHg). **(C)** Pulse rate (bpm).

**
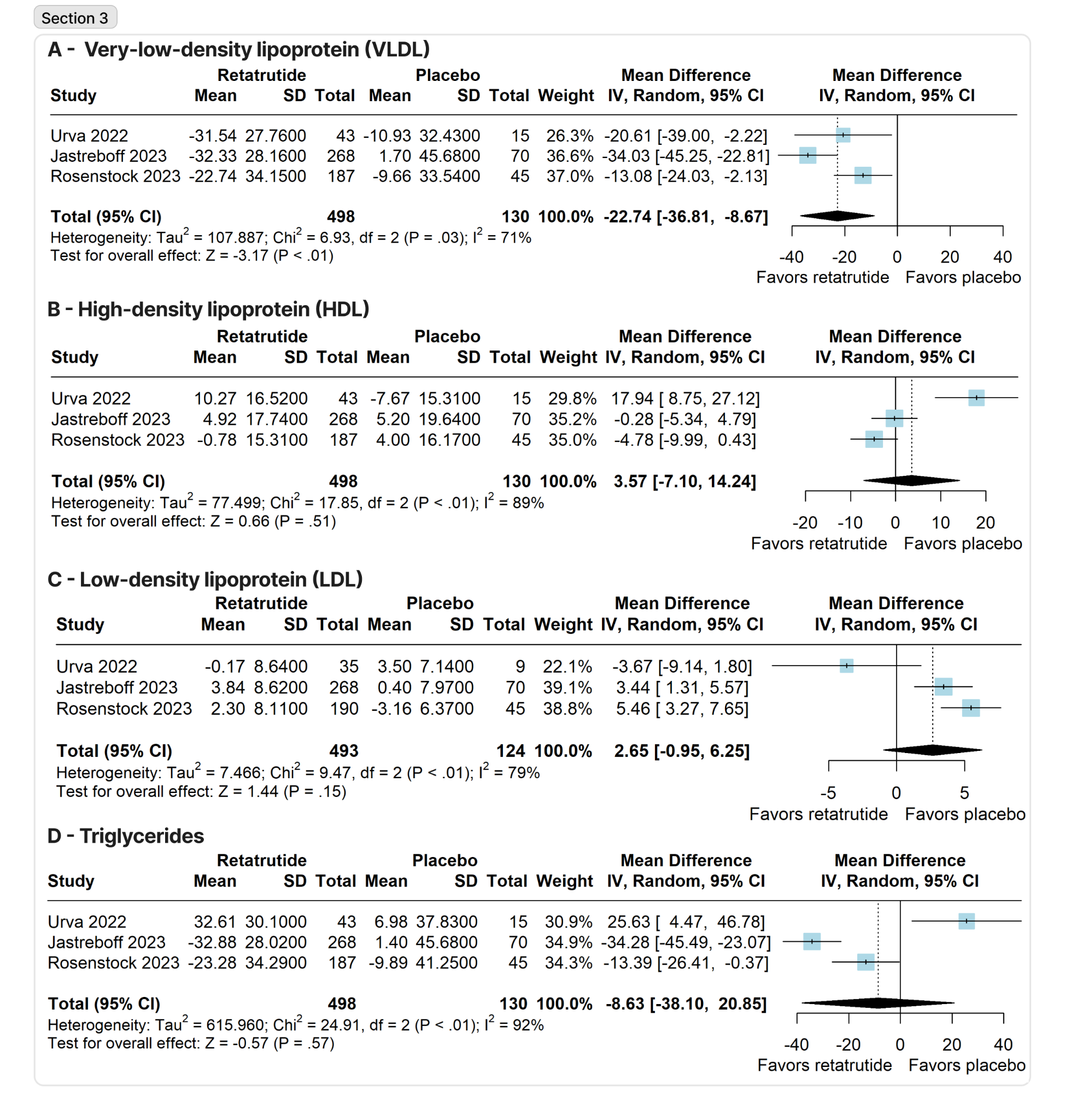
**

**Figure S3.** Forest plots of pooled comparisons between retatrutide and placebo. **(A)** Very-low-density lipoprotein (VLDL) (%). **(B)** High-density lipoprotein (HDL) (%). **(C)** Low-density lipoprotein (LDL) (%). **(D)** Triglycerides (%).

**
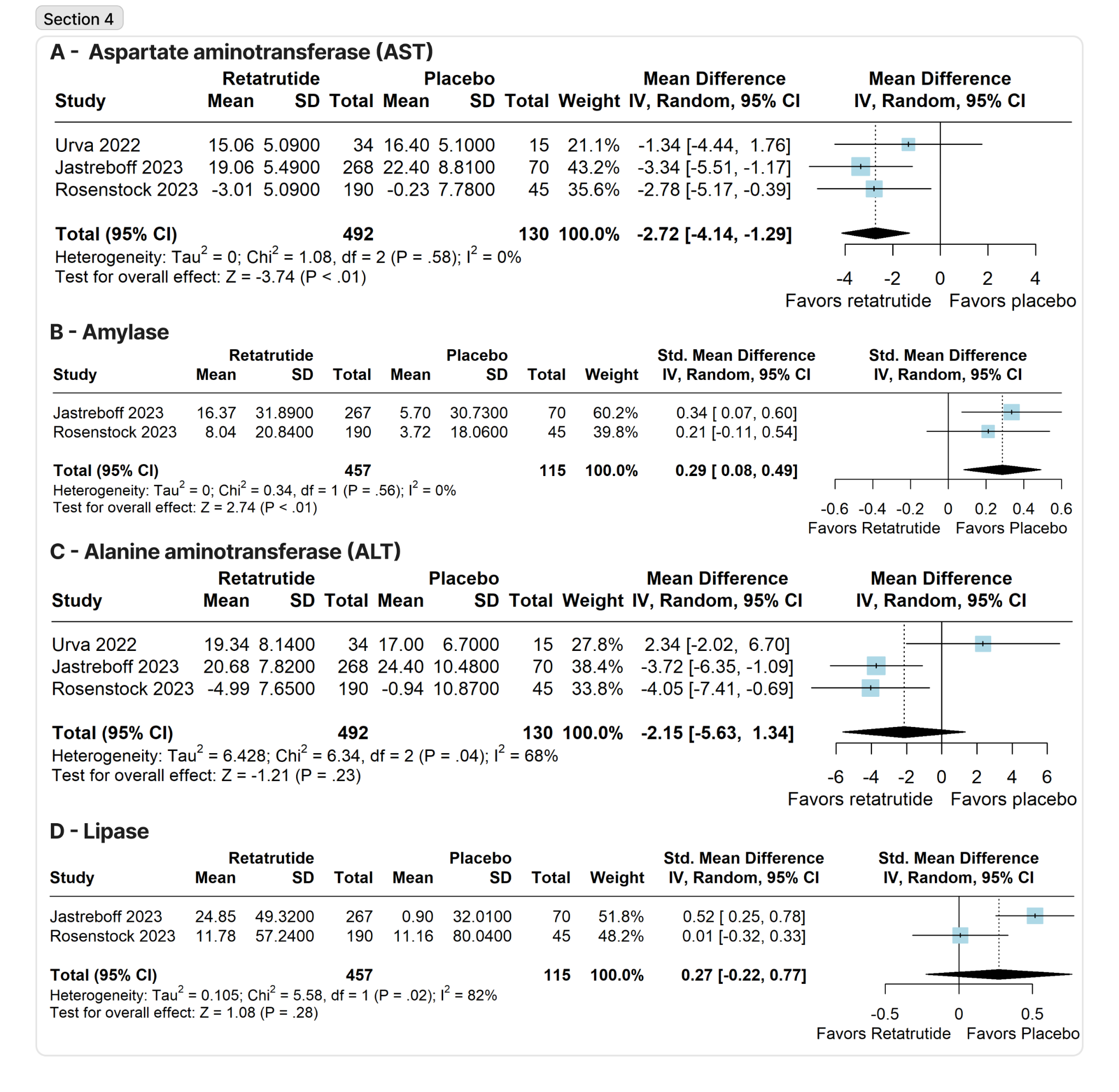
**

**Figure S4.** Forest plots of pooled comparisons between retatrutide and placebo. **(A)** Aspartate aminotransferase (AST) (U/L). **(B)** Amylase. **(C)** Alanine aminotransferase (ALT) (U/L). **(D)** Lipase.

**
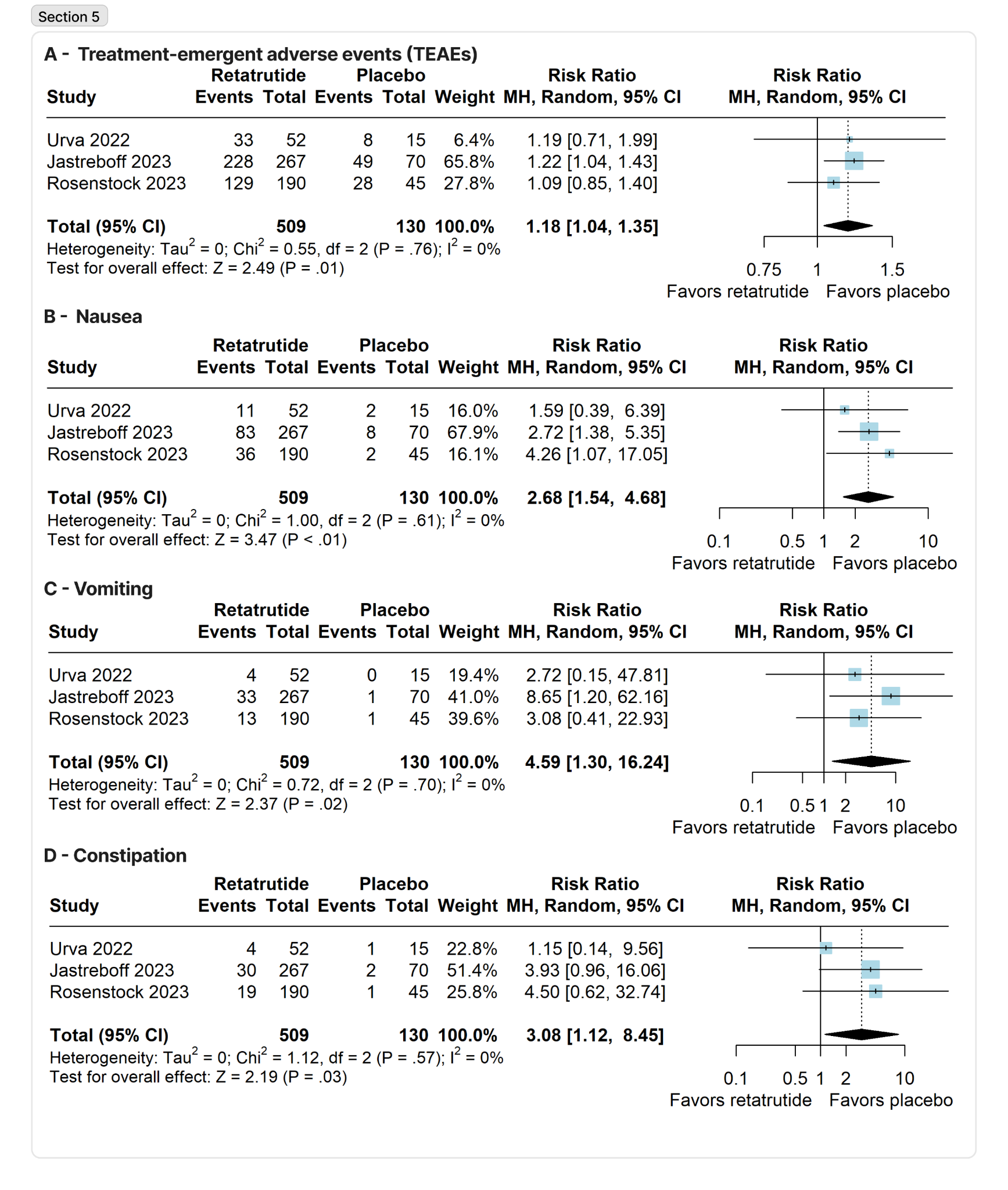
**

**Figure S5.** Forest plots of pooled comparisons between retatrutide and placebo. **(A)** Treatment-emergent adverse events (TEAEs). **(B)** Nausea. **(C)** Vomiting. **(D)** Constipation.

**
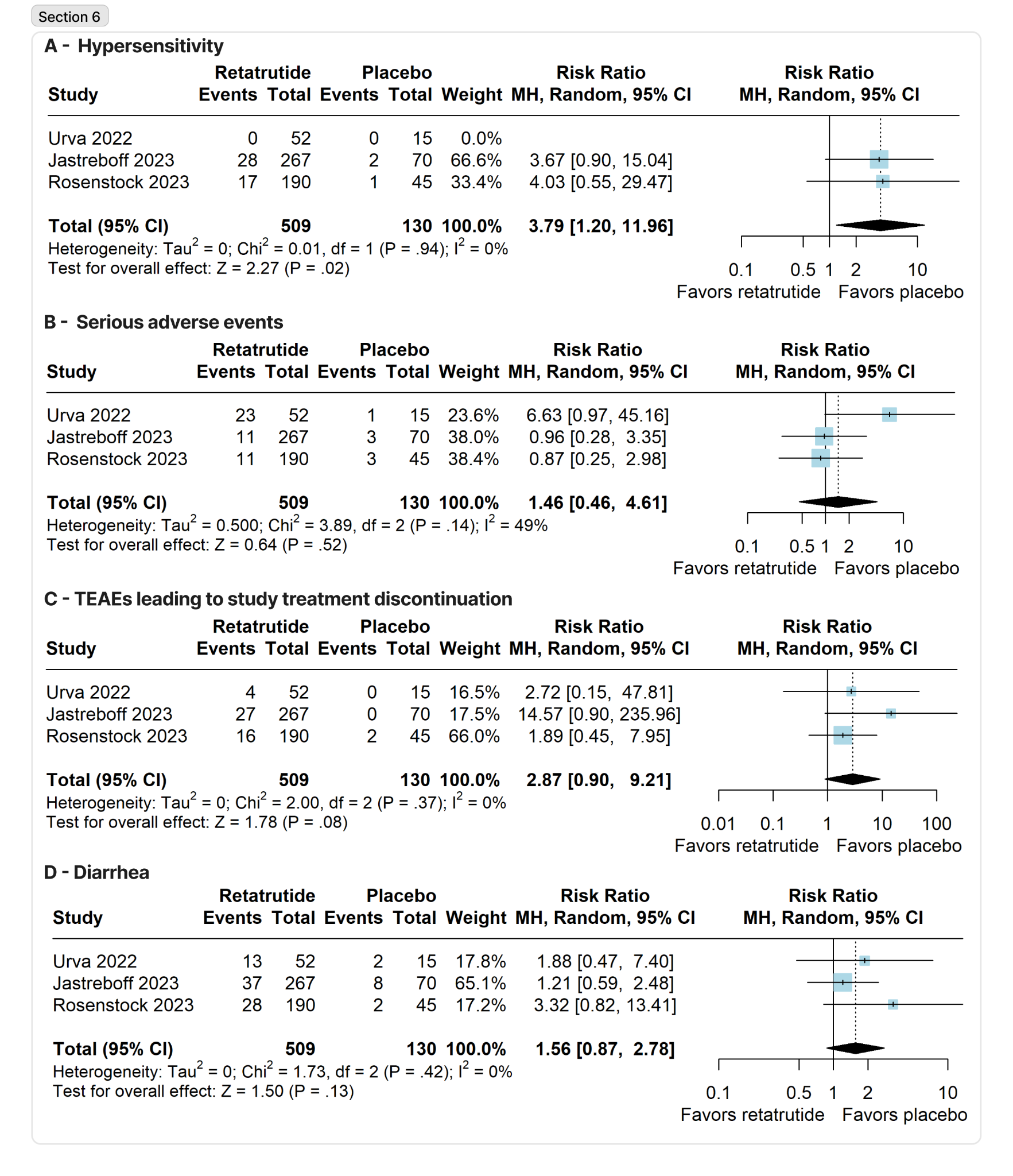
**

**Figure S6.** Forest plots of pooled comparisons between retatrutide and placebo. **(A)** Hypersensitivity. **(B)** Serious adverse events. **(C)** Treatment-emergent adverse events (TEAEs) leading to study treatment discontinuation. **(D)** Diarrhea.

**
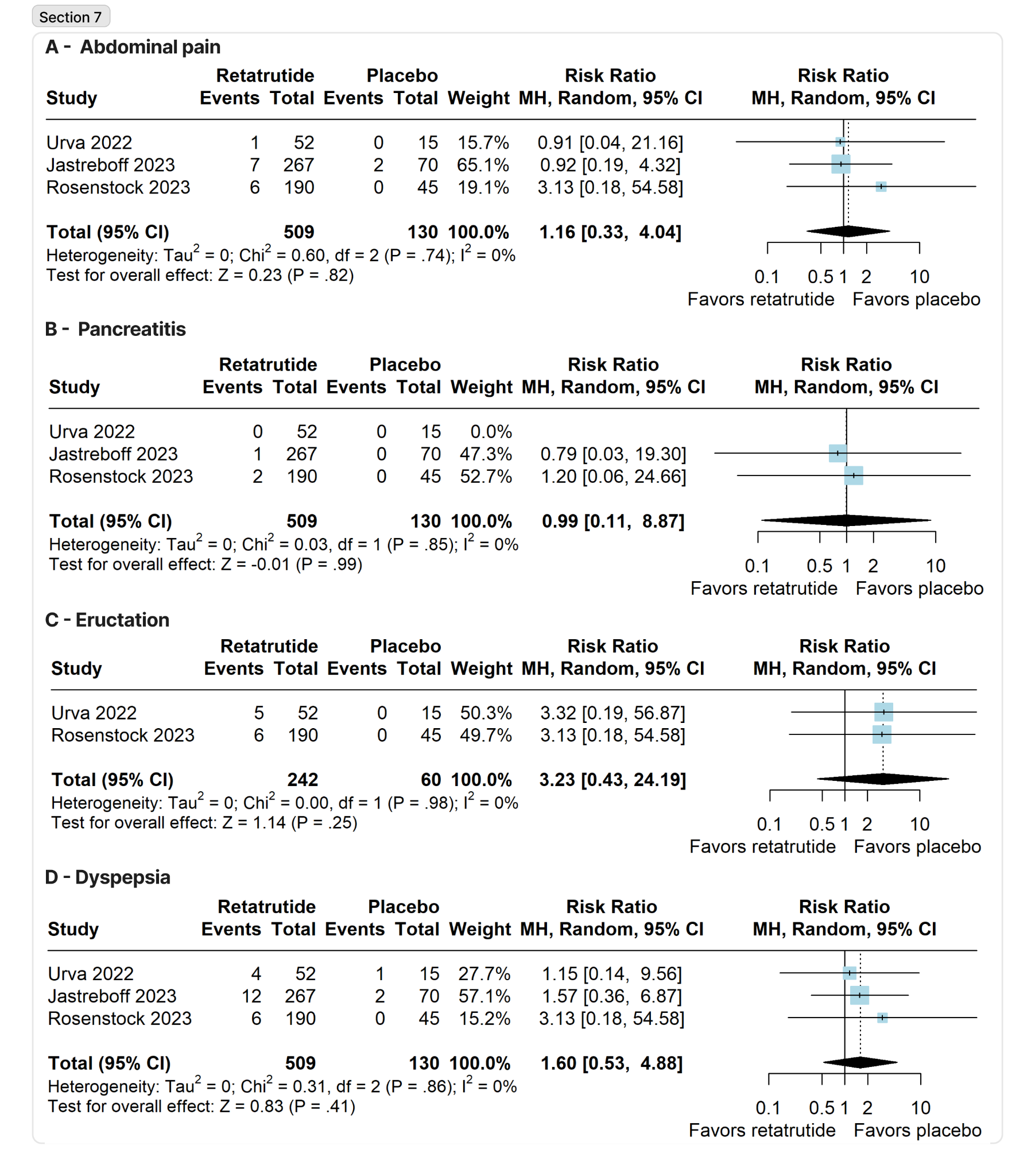
**

**Figure S7.** Forest plots of pooled comparisons between retatrutide and placebo. **(A)** Abdominal pain. **(B)** Pancreatitis. **(C)** Eructation. **(D)** Dyspepsia.

**
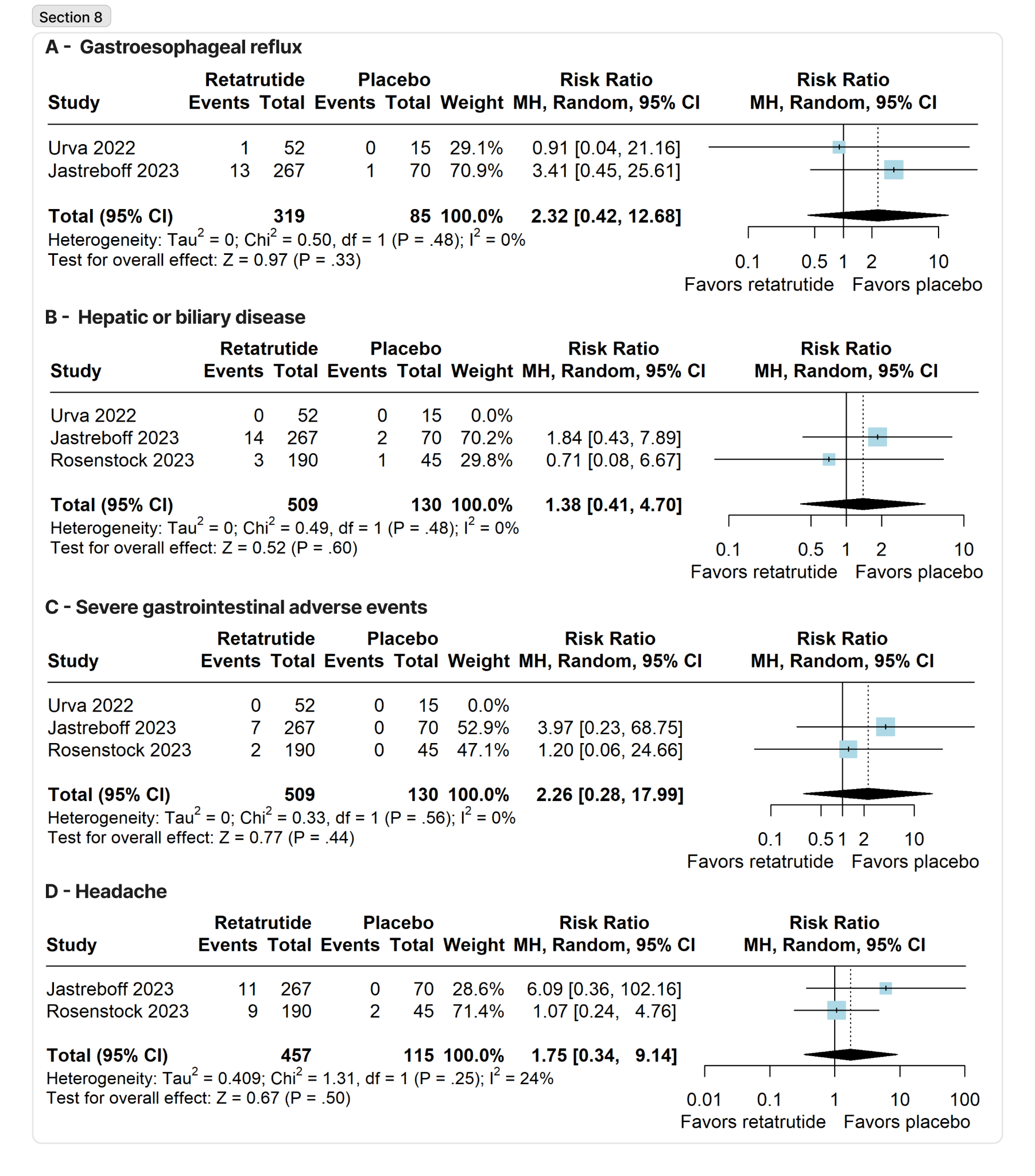
**

**Figure S8.** Forest plots of pooled comparisons between retatrutide and placebo. **(A)** Gastroesophageal reflux. **(B)** Hepatic or biliary disease. **(C)** Severe gastrointestinal adverse events. **(D)** Headache.

**
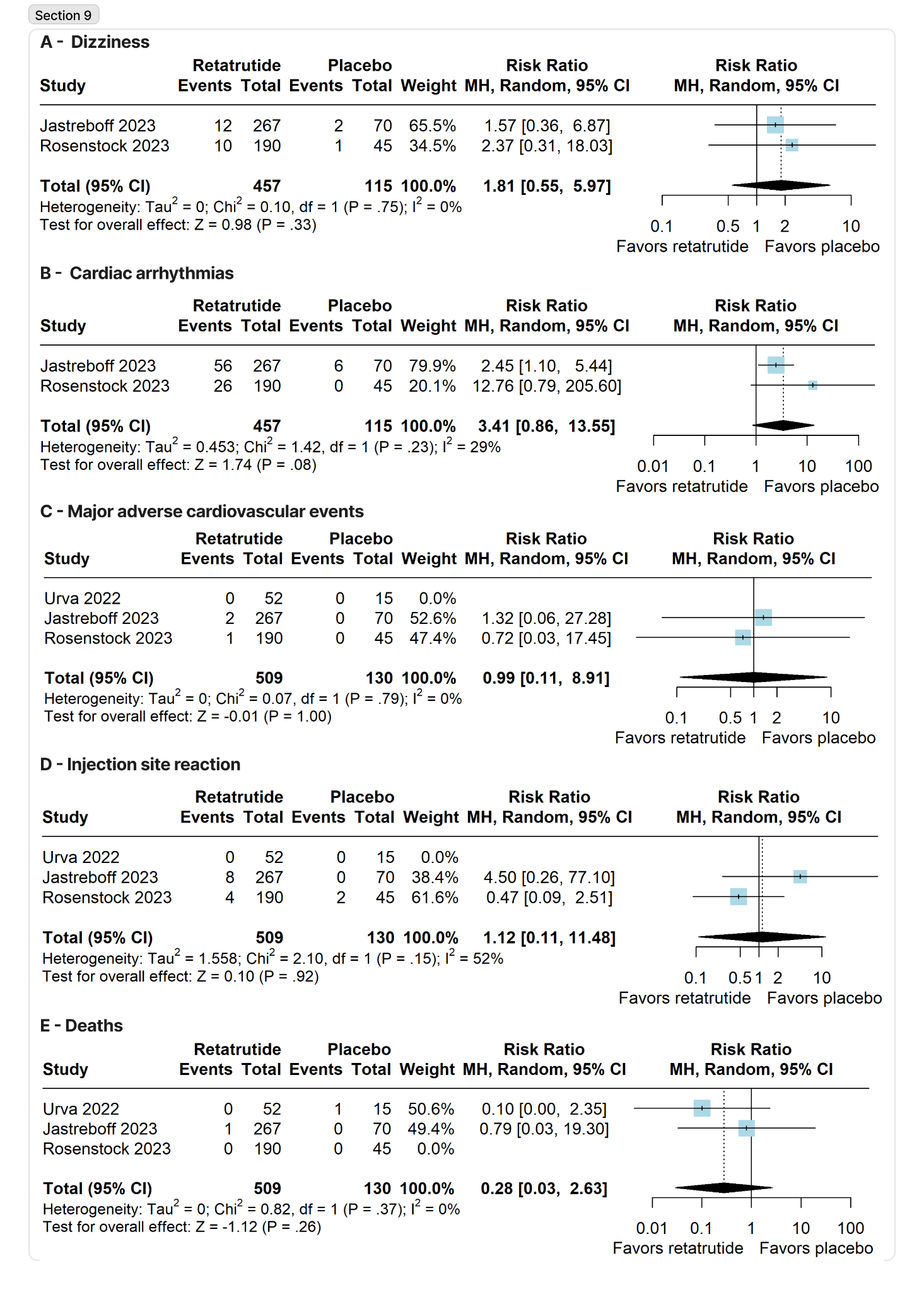
**

**Figure S9.** Forest plots of pooled comparisons between retatrutide and placebo. **(A)** Dizziness. **(B)** Cardiac arrhythmias. **(C)** Major adverse cardiovascular events. **(D)** Injection site reaction. **(E)** Deaths.

**
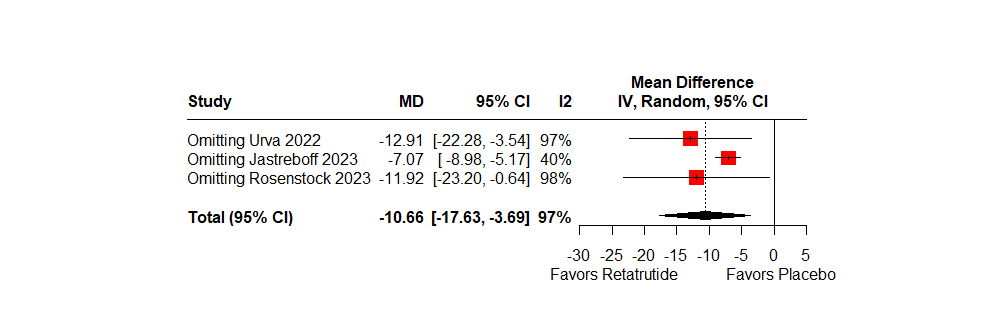
**

**Figure S10.** Leave-one-out sensitivity analysis plot for the body weight outcome.

**
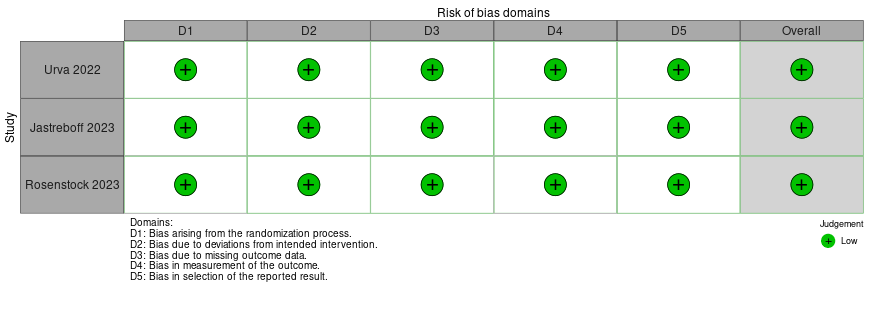
**

**Figure S11.** Critical appraisal of randomized controlled trials according to the Cochrane Collaboration tool for assessing risk of bias in randomized trials.
