# Supplementary material for: Effects of once-weekly subcutaneous retatrutide on weight and metabolic markers: A systematic review and meta-analysis of randomized controlled trials
Source: Metabol Open. 2024 Sep 13;24:100321. doi: 10.1016/j.metop.2024.100321 (PMC11420505; doi:10.1016/j.metop.2024.100321)
Supplement: Multimedia component 2 [file mmc2.pdf]

Effects of Once-Weekly Subcutaneous Retatrutide on Weight and Metabolic Markers: A Systematic Review and Meta-analysis of Randomized Controlled Trials

Bibliography: Pasqualotto et al.

Certainty assessment							Summary of findings				
Participants (studies) Follow-up	Risk of bias	Inconsistency	Indirectness	Imprecision	Publication bias	Overall certainty of evidence	Study event rates (%)		Relative effect (95% CI)	Anticipated absolute effects	
							With placebo	With Retatrutide		Risk with placebo	Risk difference with Retatrutide
**Body weight (kg)**											
625 (3 RCTs)	not serious	serious[a,b]	not serious	serious[c]	none	⊕⊕◯◯ Low	130	495	-		MD **10.66 kg lower** (17.63 lower to 3.69 lower)
**Body mass index (kg/m^2)**											
567 (2 RCTs)	not serious	serious[a,b]	not serious	serious[d]	none	⊕⊕◯◯ Low	115	452	-		MD **4.53 kg/m^2 lower** (7.51 lower to 1.55 lower)
**Weight reduction of ≥5%**											
567 (2 RCTs)	not serious	serious[a]	not serious	serious[d]	strong association	⊕⊕⊕◯ Moderate	32/115 (27.8%)	369/452 (81.6%)	**RR 2.92** (2.17 to 3.93)	278 per 1.000	**534 more per 1.000** (from 326 more to 815 more)
**Weight reduction of ≥10%**											
567 (2 RCTs)	not serious	serious[a]	not serious	serious[d]	very strong association	⊕⊕⊕⊕ High	7/115 (6.1%)	272/452 (60.2%)	**RR 9.32** (4.56 to 19.06)	61 per 1.000	**506 more per 1.000** (from 217 more to 1.000 more)
**Weight reduction of ≥15%**											
567 (2 RCTs)	not serious	serious[a]	not serious	serious[d]	very strong association	⊕⊕⊕⊕ High	3/115 (2.6%)	217/452 (48.0%)	**RR 18.40** (6.00 to 56.42)	26 per 1.000	**454 more per 1.000** (from 130 more to 1.000 more)
**Weight reduction of ≥20%**											
567 (2 RCTs)	not serious	serious[a]	not serious	serious[d]	very strong association	⊕⊕⊕⊕ High	2/115 (1.7%)	146/452 (32.3%)	**RR 16.61** (4.17 to 66.12)	17 per 1.000	**271 more per 1.000** (from 55 more to 1.000 more)
**HbA1c (%)**											
625 (3 RCTs)	not serious	serious[a,b]	not serious	serious[c]	none	⊕⊕◯◯ Low	130	495	-		MD **0.9 % lower** (1.63 lower to 0.17 lower)

CI: confidence interval; **MD:** mean difference; **RR:** risk ratio

Explanations

a. Heterogeneity between studies due to differences in baseline patient characteristics.
b. High heterogeneity.
c. Only 3 studies were included in the pooled analysis.

d. Only 2 studies were included in the pooled analysis.
